# Voice modulatory cues to structure across languages and species

**DOI:** 10.1098/rstb.2020.0393

**Published:** 2021-12-20

**Authors:** Theresa Matzinger, W. Tecumseh Fitch

**Affiliations:** ^1^ Department of Behavioral and Cognitive Biology, University of Vienna, 1030 Vienna, Austria; ^2^ Department of English, University of Vienna, 1090 Vienna, Austria

**Keywords:** voice modulation, prosody, linguistic structure, cross-species comparisons, cross-linguistic comparisons, speech segmentation

## Abstract

Voice modulatory cues such as variations in fundamental frequency, duration and pauses are key factors for structuring vocal signals in human speech and vocal communication in other tetrapods. Voice modulation physiology is highly similar in humans and other tetrapods due to shared ancestry and shared functional pressures for efficient communication. This has led to similarly structured vocalizations across humans and other tetrapods. Nonetheless, in their details, structural characteristics may vary across species and languages. Because data concerning voice modulation in non-human tetrapod vocal production and especially perception are relatively scarce compared to human vocal production and perception, this review focuses on voice modulatory cues used for speech segmentation across human languages, highlighting comparative data where available. Cues that are used similarly across many languages may help indicate which cues may result from physiological or basic cognitive constraints, and which cues may be employed more flexibly and are shaped by cultural evolution. This suggests promising candidates for future investigation of cues to structure in non-human tetrapod vocalizations.

This article is part of the theme issue ‘Voice modulation: from origin and mechanism to social impact (Part I)’.

## 1. Introduction

Although human speech is often thought to be categorically different from non-human animal vocal communication, many aspects of human acoustic communication are directly comparable with those of other land vertebrates. These include both the vocal apparatus itself and the main voice modulatory cues involved in vocal production.^1^ In this review, we will argue that voice modulatory cues are similar in the vocal communication of humans and other tetrapods because of (i) shared ancestry, resulting in a similar voice modulation physiology, and (ii) shared functional bases, i.e. similar pressures for efficient communication, resulting in similar cognitive processing due to domain-general mechanisms shared among species.

Voice modulatory cues that are shared and have similar functions in human and non-human tetrapod vocalizations as well as cross-linguistically can be hypothesized to result from anatomical, physiological and cognitive mechanisms that are evolutionarily conserved [4–6]. These include vocal tract anatomy or respiratory constraints, along with domain-general learning constraints and/or cognitive production and perception constraints (e.g. attention and memory; [4,7,8]). By contrast, cues that are neither paralleled in other tetrapods' vocalizations nor cross-linguistically varied may rely on less evolutionarily conserved mechanisms and therefore have larger potential to be shaped by cultural evolutionary processes. For example, the learnability and transmissibility of vocal features to future generations of signallers may not only be influenced by general mechanisms such as how easily the vocal features can be processed, but also by the social environment [9–14]. Thus, factors such as group identity, community size or prestige may lead to different conventions of voice modulatory patterns in different communities [15,16]. In this review, we attempt to begin disentangling which voice modulatory cues are the result of physiological constraints, of domain-general cognitive mechanisms, and of species- or language-specific conventions and learning pressures, aiming to contribute to the understanding of voice modulation in general evolutionary and cognitive terms.

Because this is a very large research program, our review will cover only some specific aspects of voice modulation. In the first section, we compare different voice modulatory cues across human speech and tetrapod communication, including pauses, fundamental frequency and syllable/unit duration. We discuss similarities and differences in the physiological mechanisms underlying these cues, and then discuss how the effort of producing and perceiving them may be linked to functional pressures in the environment. In the second part of the review, we take a comparative approach across languages, comparing whether different voice modulatory cues used for speech segmentation are similar between or differ among various human languages. Especially regarding the many voice modulatory cues for which animal data remain scarce, comparisons between different human languages may provide valuable insights as to whether the physiological and cognitive mechanisms behind those cues are species-typical (and therefore may be evolutionarily conserved and domain-general), or more flexible language-specific. Finally, our review will identify research gaps and suggest avenues for further work that may help more clearly reveal the underlying physiological and cognitive mechanisms underlying the realization of different voice modulatory cues.

Overall, our comparison between voice modulatory cues in tetrapod vocalizations and across various human languages will show that biological evolution can constrain cultural evolution, and that many of the structures and cues widely used in human speech rely upon basic acoustic and cognitive mechanisms that humans share with other tetrapods.

## 2. Voice modulation physiology and constraints on vocal production

Humans and other tetrapods share many similarities in the physiological mechanisms used to produce vocal signals. Multiple similarities result from shared respiratory mechanisms, which in turn result from shared ancestry during biological evolution [17,18]. Most tetrapods, including humans, produce vocal signals in a two-stage process: first, a **source** generates acoustic energy using an airflow from the lungs. This source is the larynx in most tetrapods and the syrinx in birds, and consists of vibrating tissue that creates sound by oscillating at a particular rate termed the fundamental frequency (*f*_0_ hereafter). This source signal is then **filtered** in the supralaryngeal vocal tract (upper respiratory tract) via multiple formant frequencies that act as a series of bandpass filters, attenuating or enhancing certain frequency ranges. The actual vocal output fuses these two components (source and filter), which are mostly independent, meaning that *f*_0_ can freely vary independent of formants and vice versa. This process, summarized as the source-filter-theory of vocal production, is shared by humans and most other tetrapods [1,19–21], with the exception of toothed whales [22] and certain whistle vocalization (e.g. in rodents; [23]). This shared physiological basis of vocal production leads to many similarities in both the production and the acoustic output of humans and other tetrapods. Nonetheless, while constrained by physiological production mechanisms, voice modulatory cues can to a certain extent be flexible, and dynamic modifications of particular acoustic parameters can provide structure to the vocal output. Specific voice modulatory cues and the extent to which they can vary (table 1) are reviewed below. In particular, we focus mainly on three cues that are well-investigated with regard to speech segmentation across human languages and will therefore be most relevant for the later sections of this review: pauses, pitch and durational cues.

**Table 1.** Voice modulatory cues in human and non-human tetrapod vocal signals, including the physiological factors that constrain them, and the specific ways in which they vary.

shared voice modulatory cues in human and non-human tetrapod vocal signals	constrained by	variation
pauses	lung capacities, respiration	number, duration, position
fundamental frequency (pitch)	subglottal pressure, length of vibrating tissue	magnitude; location of modulation
duration of syllables/units	lung capacities, respiration	magnitude; location of modulation
intensity/amplitude (loudness)	effort with which air is pushed from the lungs	magnitude; location of modulation
voice quality: formants, overtones and spectral envelope	physiology of the vocal tract, flexibility to move articulators	different sound qualities (timbre) and speech sounds (e.g. vowels)
voice quality: glottal pulses	shape of the vibrating tissue, effort with which air is pushed from the lungs	manner of vibration and shape of the glottal pulses (e.g. breathy voice)

### (a) The physiology of pauses

Among the most distinctive voice modulatory cues are pauses in the vocal signal, which often result from the need to breathe via alternating between exhaling and inhaling. Typically, tetrapods vocalize during exhalation, and vocalizations pause during inhalation. However, some non-human tetrapods vocalize during both exhalation and inhalation, and thus do not need to pause during vocalization (e.g. donkey braying, chimpanzee pant hoots or howler monkey howling, during which inhaling vocalizations are shorter than exhaling vocalizations, but similar in terms of structure and amplitude; [24]). Humans are also capable of ingressive vocalizations such as gasps and chuckles, but these usually do not replace respiratory pauses and are less flexible in encoding meaning than egressive vocalizations [25–27]. While pauses in tetrapods result from the same physiological mechanism, i.e. respiratory pausing, and are thus constrained by the individuals’ lung capacities, they can also vary in their specific realizations. For example, pauses can differ in their duration, number and their position in the vocal stream. Because of this flexibility, tetrapods, including humans, can use pauses to structure the vocal signal in many different ways [28]. For example, birdsong is structured into units commonly termed ‘syllables’ that are separated by short pauses during which rapid inhalation—‘mini-breaths’—occur [29].

### (b) The physiology of duration

The duration of phonation at the source can induce durational and rate variations in the vocal output. These durational variations can extend over different domains of the vocal output, such as individual sounds, individual syllables/units or larger stretches of vocalizations [30,31]. This can, for example, lead to different rhythmic patterns, to differences in vocalization tempo, or to distinctive vowel sounds in human speech, where phonemic distinctions between long and short durations are frequent. Duration of one syllable can also disambiguate neighbouring phonemes, as exemplified in the American English words *ladder* (/æ/ longer) and *latter* (/æ/ shorter), which only differ in their vowel length [32]. Human speech sounds that differ in their vowel quality (determined by formants), such as the vowels in the English words *feet* and *fit*, may also have distinctive lengths. Again, physiologically, durational variations are limited by the individuals' breathing capacities, but below that capacious limit, the duration can be varied more or less flexibly to give structure to the vocal output of humans and most non-human tetrapods alike.

### (c) The physiology of pitch

Vocal signals are further characterized by the vibration rate of the vibrating tissue, which determines the signals’ *f*_0_, often termed pitch in the speech literature [21]. Typically, in tetrapods, *f*_0_ is influenced both by subglottal air pressure and by muscles that regulate the length and tension of the vibrating tissues, i.e. the vocal folds in non-avian tetrapods and the syringeal membranes in birds [33–35]. By modulating these two factors, a pitch can vary within and between vocal signals. To increase pitch, individuals can either increase the subglottal air pressure or the tension of the vibrating tissues. Both of these options require increased effort (see §3) and can provide diversity and structure to vocal signals. For example, typically, on the level of syllables, an increase in pitch signals emphasis (‘stress’ in the speech literature), whereas pitch modulation on the phrase level can function as a boundary signal [36–39]. Again, the effort required for pitch modulation, and physiology such as the dimensions of the vibrating tissues, limit the pitch range that can be realized. However, within that range, the pitch can be employed flexibly to structure the vocal signal differently, as evidenced by different stress patterns observed in different languages [40].

Fundamentally, tetrapods share these voice modulatory cues because of their shared vocal production physiology, which in turn results from their shared ancestry. Nonetheless, the specific uses and manifestations of these cues can vary considerably across species and languages. For example, species, languages and individuals may differ in when and where they make pauses, when and where pitch rises and falls, or which segments they lengthen or shorten. One useful principle for categorizing and understanding this variation in vocal signals is based on the effort it takes to place emphasis in the vocal signal, using various voice modulatory cues. Thus, the following section will address emphasis and effort in the production of vocal signals, how they are influenced by functional pressures and how this can lead to the cultural evolution of prosodic patterns.

## 3. Emphasis and effort

It seems intuitively obvious that vocal signals can carry emphasis, and that this requires effort. In particular, it takes more effort to produce emphasized or stressed, i.e. louder, longer and higher-pitched syllables than non-emphasized or unstressed ones. However, despite a common assertion that producing certain voice modulatory cues is more ‘energetically efficient’ than producing others [41–45], the exact metabolic costs needed to produce and process these cues have rarely been systematically compared. In fact, several studies have shown that vocalizing is not very costly in terms of oxygen, glucose or ATP needed [46–50]. Thus, although it is clear that tensing muscles requires energy consumption, the costs involved in contracting the tiny muscles controlling source characteristics like *f*_0_ may not be appreciable relative to an organism's overall energy budget. Respiratory muscles are larger and potentially more energy-consuming, but they need to be constantly working to serve respiratory functions, independent of vocalization. The relative cost of increased versus decreased pitch or duration during normal speech and frequently produced animal vocalizations will represent an even smaller proportion of net energy expenditure.^2^ Finally, the cost of neuronal firing involved in producing or perceiving vocalizations is real, but also very difficult to quantify using current methods. Therefore, at present, we have little choice but to adopt an intuitive definition of ‘effort’, which can manifest in dynamic effort, i.e. muscular effort for moving the articulators, and neural control effort, i.e. cognitive effort for planning, producing and processing voice modulatory cues. The term ‘stress’ is used in phonology essentially as a catch-all term, connoting effort and emphasis, but not grounded in detailed syllable-by-syllable measures of expended effort.

How much effort senders will invest in emphasizing vocalizations is largely driven by an interplay of the functional pressure for successful versus efficient communication [42,51]. These pressures may also influence which parts of the signal are emphasized. Emphasis can either extend over the whole signal (e.g. louder vocalizations in noisy environments) or be specific to certain elements of the signal (e.g. stressing certain phrases or syllables); the latter should be more energetically efficient, so we may expect organisms to vary cues across a vocal stream in many cases, as humans do with speech.

One well-studied example where signals are emphasized in their entirety is the so-called Lombard effect: both humans and other tetrapods, including non-human primates, birds and whales tend to vocalize louder and with a higher pitch, i.e. with an increased effort, when there is more background noise [52–56]. When background noise in the environment is reduced, signallers return to vocalizations that need less effort and decrease their pitch and intensity. A recent example in birdsong occurred when traffic reductions during the Covid-19 pandemic resulted in lower-frequency bird vocalizations, showing that signallers can flexibly adapt their vocalizations to functional pressures in the environment [57].

Further examples of signals with emphasized elements include rhythmic vocalizations and stress or intonation patterns. This kind of emphasis needs both dynamic and cognitive effort on the side of the sender, but creates structure in the signal, which may reduce error, combat habituation or facilitate meaning encoding and processing on the side of the listener [58]. The complex interplay of pressures acting on the sender and receiver may lead to variation in vocal signals that is not fixed genetically but influenced by current properties of the environment [9,10] and shows that once individuals begin to produce vocal cues, there is an opportunity to modulate them. Furthermore, in species that learn their vocalizations (e.g. birdsong or human speech), small production or perception biases for or against certain voice modulatory structural patterns in a certain environment may be amplified over generations of speakers [9]. This may lead to a process of cultural evolution and can result in within-species variation in structural patterns of vocalizations as exemplified by different human languages or different dialects in other tetrapods' vocalizations [59,60].

Thus, overall, how exactly the different voice modulatory cues are used varies within physiological constraints and results from a balancing act between communicating successfully, but with low effort. This in turn depends on functional pressures of listeners and environment, which can vary between different species and languages, and may include factors such as cultural evolution. How exactly different species and different linguistic communities deal with different functional pressures depends both on domain-specific factors such as auditory salience, domain-general cognitive constraints such as memory and attention, but also on more flexible constraints such as social factors. All of these factors will combine to constrain the range within which the different voice modulatory cues can be realized and determine the actual vocal output seen in a language or a species.

## 4. What we can learn from comparing voice modulatory cues across human languages

Different realizations of voice modulatory cues have been heavily investigated in human languages, but similar investigations in non-human tetrapod vocalizations are comparatively scarce and less systematic. Over the past decades, bioacoustics has made considerable advances in the investigation of non-human tetrapod vocal production, but research on the perception of voice modulatory cues in non-human tetrapods is still in its infancy [61,62]. It is especially difficult to reach firm conclusions about the communicative meaning of voice modulatory structures found in non-human tetrapod vocal signals, given how few cues and species have been systematically investigated.

Therefore, the remaining sections of this review will mainly focus on the comparison of voice modulatory cues across human languages, and specifically the voice modulatory cues that help listeners to segment continuous speech into words. When voice modulatory cues are realized similarly across human languages, this suggests that fundamental physiological constraints or basic cognitive mechanisms may be responsible for these patterns [4–6], and that therefore, due to their shared ancestry, similar cues may also be prevalent in non-human tetrapod vocalizations. We suggest that such patterns may provide starting points for investigating modulation in tetrapod vocal signals. By contrast, cues that differ across different linguistic communities may be largely influenced by different functional pressures in the environment and by cultural evolutionary processes and therefore are more likely to also differ across tetrapod vocalizations.

Comparing voice modulation across human languages and non-human animal vocalizations, and using similarities and differences between them to draw conclusions about the evolutionary roots of vocal communication, is not new [2,63–66]. Similar approaches have already been proposed, for example, by Morton [64,65], who suggested that high and low pitch vocalizations signal similar emotions and attitudes across languages and species. Across species, a low pitch signals largeness, dominance and self-confidence, whereas a high pitch signals smallness, submissiveness and prosociality. Ohala [67] suggests that this biological grounding helps to explain prosodic patterns that are consistent across human languages, such as a final pitch decrease in declarative statements (i.e. utterances signalling dominance and self-assurance) and final pitch increase in questions (i.e. utterances signalling insecurity, submissiveness and need).

Past approaches typically either avoid detailing the specific acoustic cues [66], or treat these cues as fixed for a particular sound class (e.g. low-pitched growls and high-pitched whines). Our goal below is to call attention to how dynamics *within* a call can play a role in structuring acoustic signals, and to investigate the specific acoustic parameters varied. Furthermore, our approach extends previous proposals by highlighting the importance of listener-associated cognitive factors, such as perceptual salience, memory, attention and learnability of prosodic patterns, for biological and cultural evolution. Finally, our proposal captures a more diverse range of prosodic patterns than previous accounts. In contrast with Ohala [67], who explained prosodic patterns by primarily drawing on emotional communication, our account attempts to explain a more diverse set of linguistic structures and meanings.

## 5. Structure in human languages: the speech segmentation problem and cues to solving it

One crucial first step in the acquisition of linguistic structure is the segmentation of fluent speech into words, before the words’ meaning is known. This so-called speech segmentation problem is most acute for infants learning their first language, but also concerns second language learners. For adults, the challenge is particularly evident when they try to identify distinct words while listening to an unfamiliar foreign language [68–70]. Nevertheless, language learners eventually master the speech segmentation problem easily. This is because they implicitly use various cues in the speech stream to identify patterns and regularities, which in turn help them to extract words. Such cues may also play a role in complex sequence learning in bird or whale song (e.g. [71]), but this possibility remains little explored.

Speech segmentation is a challenge that speakers of all human languages have to face and that is therefore well suited for cross-linguistic comparisons. Over the past decades, cues used in human speech segmentation have been the subject of a large body of research in a variety of different languages such as English [72–76], German [77–80], Italian [78,79,81], French [74], Dutch [74], Spanish [79,82], Portuguese [83], Basque [79], Japanese [73], Cantonese, Mandarin and Russian [84]. This makes it possible to compare the characteristics of speech segmentation cues across languages, answer questions about more general physiological and cognitive mechanisms that are necessary to create and process linguistic structure and identify functional pressures in the respective environments. Among the cues that have been identified to be very important for speech segmentation and creating linguistic structure are transitional probability cues (statistical learning) and the voice modulatory cues that are our focus (e.g. [68,73–75,85–92]).

Transitional probability cues are based on listeners tracking the co-occurrence frequencies of syllables in vocal input ([75,93]; see [94] for a meta-analysis). For example, when hearing the sound sequence *pretty#baby*, listeners can infer that *pretty* and *baby* are distinct words because the syllables *pre* and *ty* as well as *ba* and *by* also co-occur in other sequences such as *pretty#girl* or *lovely#baby*. By contrast, *ty* and *ba* co-occur less frequently and can therefore be assumed to span a word boundary [95]. Speakers of a wide variety of languages have been demonstrated to use such transitional probability cues for language acquisition in similar ways (English: e.g. [72–76]; German: [77–79]; Italian: [78,79,81]; French: [74]; Dutch: [74]; Spanish: [79,82]; Portuguese: [83]; Basque: [79]; Japanese: [73]). Notably, producing different speech sounds and syllable identities is itself a form of voice modulation and is a prerequisite for syllable creation and thus for tracking transitional probabilities. Specifically, individual vowels and consonants are created by moving the articulators, which leads to different formant frequency patterns (see table 1; [96]). While different languages have different speech sounds [40,97], the cross-linguistic ability to modulate the voice in a way that produces different speech sounds is crucial for the cross-linguistic use of transitional probabilities for speech segmentation.

Using transitional probabilities to infer characteristics of a signal appears to be a very general behaviour since in basically any domain of action, including animal vocalizations, certain events are more likely to follow each other than others [98,99]. In humans, the identification of transitional probability cues appears to be based on a domain-general cognitive mechanism, namely statistical learning [100–103]. Furthermore, statistical learning is not a uniquely human cognitive mechanism, and also other species have been demonstrated to use it to deduce signal structure [104]. These can even apply across species; for example, many non-human animals form associations between heterospecific alarm calls and the presence of a predator [105,106]. Also, vocal learning in non-human animals, most notably in birds, is suggested to be supported by statistical computations, although the precise mechanisms behind it are not yet fully understood [104]. It thus seems likely that both humans and many non-human tetrapods rely on a combination of statistical learning and acoustic modulations when learning the structure of their species-specific sound sequences.

Statistical learning is a very general and prominent perceptual and cognitive skill. However, in human languages, voice modulatory cues in the speech stream, such as pauses, or variations in fundamental frequency, syllable duration or intensity (which create word stress, speech rhythm or intonation), can be processed more easily than statistical cues and therefore have more significant effects on speech segmentation [68,76,80,81,91]. However, since voice modulatory cues come in many different realizations and can have many different functions [107], their overall role in signalling linguistic structure, and the cognitive mechanisms needed for processing them, are less understood. While some voice modulatory cues are realized and processed similarly across languages (e.g. [73]), others are subject to cross-linguistic variation (e.g. [74,79]). This raises the question how much the realization and processing of voice modulatory cues are determined by domain-general cognitive or physiological constraints, and how much these cues may be shaped by cultural evolution.

## 6. Cues to speech perception: when voice modulatory cues count more than transitional probability cues

The efficiency of different voice modulatory cues for speech segmentation has traditionally been tested in artificial language learning experiments [75]. In these experiments, participants are exposed to several minutes of a continuous stream of nonsense speech, consisting of randomly concatenated invented pseudo-words. Listeners can infer from the transition probabilities between syllables which syllable combinations are ‘words’ of the artificial language and can segment these items from the stream. To test the influence of voice modulatory cues on listeners' segmentation performance, voice modulatory cues are added at different positions to the speech stream and it is measured how this changes listeners’ perception of words in the stream.

In such artificial language learning experiments, voice modulatory cues added to continuous speech on the word (e.g. [73,74,80]) and phrase level (e.g. [108–111]) typically enhance speech segmentation compared to transitional probability cues only. Crucially, these cues facilitate speech segmentation most effectively when they converge with the transitional probability cues in the speech stream, i.e. when the voice modulatory cues sound as ‘natural’ to the listeners as they do in natural speech. By contrast, when voice modulatory cues are designed to conflict with the transitional probability cues in experimental settings and sound ‘unnatural’ to the listeners, voice modulatory cues disrupt speech segmentation or even override the transitional probability cues [68,76,80,81,91]. Whether voice modulatory cues at certain positions in the speech stream sound natural or unnatural with respect to the transitional probability cues depends both on language-universal cognitive predispositions such as attention, perception or preferences in pattern recognition, and on language-specific word stress patterns typical of the listeners' native languages [73,74,81].

Crucially, many artificial language learning studies tested the influence of language-specific word stress on speech segmentation by using a combination of different voice modulatory cues [74,78,81]. For example, stress cues dominated transitional probability cues when they were implemented as a combination of longer-duration, higher-pitch and higher-intensity of stressed syllables [68,76,91]. While using a combination of different voice modulatory cues closely simulates natural languages [70,91,92], it does not tell anything about the effects of the individual voice modulatory cues in isolation. However, since different voice modulatory cues have different physiological origins and may be cognitively processed and culturally transmitted differently, investigating them separately can reveal more about the functional pressures acting on linguistic structure [81,88].

Several studies have already addressed the role of voice modulatory cues in isolation. These studies suggest that pauses and lengthening serve as language-universal signals for word-finality (e.g. [73,74,78,79,85,88,112]; but also: [81,113]). By contrast, pitch increase is suggested to be the main perceptual correlate of word stress and is therefore processed differently by speakers of different languages [68,74,78,114]. Speech segmentation studies investigating other prosodic cues such as intensity or voice quality are comparatively rare [88,115], which is why our review below focuses on pauses, durational and pitch modifications.

## 7. Pauses

Pause cues typically result from the physiological necessity to breathe, but pauses could in principle be expressed at different positions in a vocal signal, or differ in number and duration. Still, in practice, pauses are realized in strikingly similar ways across human languages. Language-universally, pauses are realized at the end of sentences or phrases but hardly ever occur within phrases or within words [28,116]. This is further supported by second language learning studies finding that second language learners have hardly any problems acquiring pause characteristics typical of their second language [117,118]. Thus, while in principle, pauses could occur anywhere within the breathing range, it is most probable that domain-general cognitive processing mechanisms constrain them to occur at specific positions in the vocal output—namely at those positions where they structure the vocal output most efficiently and with the least processing effort.

This and their perceptual salience may explain why pauses are very effective for speech segmentation and outrank other cues in speech segmentation experiments [80].

In animal vocal signals, it is challenging to determine whether pauses occur between or within phrases because units and phrases in animal vocalizations are less clearly defined [119]. Still, because of their shared ancestry with humans, it can be expected that pauses manifest similarly in non-human tetrapods’ vocalizations, i.e. at the end of phrases or units. This is why pauses are often used by researchers to determine units in non-human tetrapod vocalizations [120].

## 8. Final lengthening as a cross-linguistic segmentation cue

One reason why final lengthening may serve as a language-independent speech segmentation cue is that—language-universally—sentence-final or phrase-final elements are lengthened in everyday speech production [28,74,121–124]. The evolutionary origins of final lengthening are that at sentence or phrase boundaries, speakers need to switch from exhaling to inhaling, leading to a pause, and that it takes less effort to slow articulators down before a pause than to stop them abruptly [125–129]. Similar patterns can also be observed in movements in other domains than vocalization. For example, runners also decelerate their movements before stopping [130]. This mechanistic factor seems like a good candidate for a factor that could play a role across languages and in other species' vocal communication systems: a potential universal in vocal communication.

Because kinematic articulatory constraints result in lengthened syllables before sentence or phrase boundaries, listeners may have learned to associate lengthening with boundaries and to exploit it as a cue for speech segmentation [131]. In turn, speakers may have started to intentionally use lengthening to indicate boundaries in the speech stream, also at positions where they did not pause [132]. Via cultural transmission, this may have resulted in final lengthening becoming a conventionalized but still language-universal boundary signal [133]. Because final lengthening is used as a convention for indicating boundaries cross-linguistically, it can be assumed that besides the articulatory constraints that speakers of all languages face equally, its transmission and processing is based on domain-general cognitive constraints.

This notion is supported by the putatively language-independent Iambic/Trochaic Law (=ITL; [134–138]), which states that cross-linguistically, listeners group sounds with longer duration as sequence-final (iambic grouping). Although the ITL focuses on disyllabic words, it can also be generalized to trisyllabic words, suggesting that domain-general cognitive mechanisms may be responsible for this flexibility [73,80]. Still, recently, there has also been evidence that the perceptual groupings of sequences of syllables with variable duration may be shaped more by cultural variation than previously assumed [81,139–141]. Interestingly, the ITL not only applies to linguistic stimuli, but also to tone sequences [115,137] or visual patterns [142]. This further supports the idea that final lengthening as a signal to linguistic structure and thus to low-effort communication results from general cognitive processing mechanisms that also apply to non-linguistic stimuli.

Since deceleration before pauses occurs across various human movements [130] and final lengthening is perceived as a boundary signal across different sensory domains, the mechanisms behind it seem likely to be evolutionarily old. Because of their shared ancestry with humans, a similar vocal tract physiology and similar energetic constraints, final lengthening and its perception as a boundary signal are promising targets for investigation in non-human tetrapods, and there is already some evidence for final lengthening in birdsong [143,144]. Such a cue could play an important role, for example, in structuring turn-taking exchanges between individuals [145,146]. However, to our knowledge, there is no current evidence that non-human tetrapods use final lengthening as a boundary cue at a perceptual level, and when listening to human speech, rats do not appear to group syllables varying in duration according to the ITL [138]. Research with other tetrapods is badly needed to further examine this potential universal.

## 9. Pitch cues as language-specific segmentation cues

In multiple speech segmentation experiments, similar pitch modifications led to different segmentation patterns in speakers of different native languages [74,78]. For example, word-initial pitch increase facilitated speech segmentation for native speakers of English, whereas word-final pitch increase facilitated speech segmentation for native speakers of French. These patterns are consistent with the typical stress placements of these languages [74,147].

One explanation why duration and pitch are used differently for speech segmentation is that, potentially, pitch is used as a more reliable cue for the perception of word stress than duration. In speech *production*, stressed syllables are characterized by a co-occurrence of higher pitch and longer duration, and interestingly, cross-linguistically, duration seems to be a more consistent marker of word stress than pitch ([81,148]; but also: [74] for French and English). Still, while being an important acoustic correlate of word stress, lengthening at the same time occurs at boundaries (as discussed in the previous section) and most likely, this durational increase is larger and more consistently applied than that at stressed syllables [125]. As a result, during *perception*, to avoid ambiguities, listeners may rely on lengthening for perceiving boundaries, but rather focus on the pitch for perceiving word stress [74,80].

In general, listeners may need to be more flexible in the perception and cognitive processing of pitch variations compared to durational variations. In natural speech, pitch as a signal for word stress varies more than duration as a signal for sentence or phrase finality, for example, because of loan words with non-typical stress patterns [149–151]. In addition, intonation patterns are variable and depend for example on speaker emotions, attitudes, grammatical structure and focus [152]. Also, while sentence-final pitch decrease in declarative sentences is common across languages [110,123,153], listeners may equally encounter sentence-final pitch increase in yes–no questions. Therefore, overall, the pitch may be a less consistent [41,154–156] and less informative cue during speech segmentation than lengthening. This may explain why neither word-final pitch decrease [80] nor increase facilitated speech segmentation [74,78,157] in artificial language learning experiments, unless for speakers of languages with word-final stress [74,147].

According to the ITL [136,138,158–160], listeners perceive sounds with a higher pitch in sequence-initial positions (trochaic grouping). Interestingly, rats similarly group sequences that vary in pitch as trochees [138]. However, apparently, this perceptual grouping does not play a big role for speech segmentation, since cross-linguistically, a word-initial higher pitch has facilitated speech segmentation in artificial language learning experiments only inconsistently [74,80,81,157]. It can therefore be inferred that the ITL for pitch does not systematically generalize from disyllabic to trisyllabic words, but pitch is instead processed more flexibly.

The apparently rather flexible processing of pitch may result in weak production, perception or learning biases amplifying pitch cues in different directions during the cultural transmission of languages. This may in turn lead to different stress patterns in different languages, making pitch a less reliable signal for speech segmentation than duration. While still originating from basic cognitive processing mechanisms, the cognitive and physiological structures responsible for pitch processing are therefore suggested to be less conserved than those responsible for duration processing. This may have constrained the cultural evolution of pitch cues to linguistic structure less than that of durational cues. Thus, functional pressures for structured signals may hold equally across languages, but how exactly this linguistic structure is archieved, can vary cross-linguistically.

While lexical stress patterns vary across languages and it can be assumed that similar variation should be expected in other tetrapod vocalizations, utterance-final pitch decrease in declarative statements is common across many languages [39,110,123,153]. One reason for this declination may be that the articulators, in this case the vibrating tissues, are slowed down before being brought to a halt, and this lower vibration rate of the tissues leads to a lower pitch [161]. A functional reason may be that pitch declination facilitates turn-taking and thus decreases communicative effort.^3^ These physiological and functional constraints are shared across species, which is why pitch declination may be an interesting target for investigation in non-human tetrapod vocal signals. Indeed, there are some indications for final pitch declination and turn-taking in vervet monkeys and rhesus macaques [38]. Investigating other species for final pitch declination could further corroborate the hypothesis that a shared ancestry drives similarities in pitch realization and processing in humans and non-human tetrapods.

## 10. Conclusion and outlook

Summarizing, our review of human speech modulation shows that *f*_0_, duration and pauses are typically used in systematic ways across languages to help structure the speech signal, but that there is nonetheless considerable variation across languages in the details. Voice modulation can, in many cases, provide cues to structure that are more salient and effective to listeners and learners than statistical measures over the vocal units (e.g. sequential transition probabilities), and can work together with such statistical information or in some cases override it. Thus, although such statistical cues are important (and can be readily computed in animal signals like bird or whale song), they obscure the importance of voice modulation as a key factor in structuring animal communication signals.

How language- or species-specific and cross-linguistic and cross-species cues interact certainly warrants further research. In those cases where comparative information is available, it suggests that the cues used to indicate a structure in the speech signal are both present in vocalizations of other species (unsurprising given their fundamentally similar production mechanisms) and also can be used in similar ways (e.g. phrase-final lengthening in speech and birdsong). Nonetheless, there is currently far too little comparative data to allow any clear conclusions about the degree to which human-typical cues to structure are also used by other species. More research in this area—what we might term ‘animal phonology’—is needed to evaluate whether there are broad phylogenetic generalizations to be made, as we have hypothesized here. A rich comparative analysis of these issues could be expected to shed light not just on the evolution of communication across vertebrates, but also about the phylogenetic origins of universals in human speech production and perception.

## Footnotes

1 The terms ‘voice modulation’ and ‘prosody’ essentially describe the same concept, namely all kinds of vocal dynamic modifications of acoustic parameters during production in humans and non-human tetrapods [1–3]. For the sake of consistency, we will use the term ‘voice modulation’ throughout this review.

2 Note that respiratory muscles may induce higher energetic costs in very loud, high or long vocalizations such as during human singing and oratory, or mammalian roaring contests or infrasonic long-distance calls. Because subglottal pressure is an important factor determining both *f*_0_ and sound intensity, very loud and high-pitched vocalizations may require more respiratory effort than normal breathing and vocalization. In addition, very long syllables may disrupt the natural respiratory rhythm.

3 However, potential analogies between turn taking in human and non-human animal vocalizations have to be interpreted with caution. Since it is difficult to assess the underlying meaning or the intentions behind non-human animal vocal signals, alternation of signals may not necessarily be the result of active turn-taking [146]. In such cases, the communicative benefit gained from alternating vocalizations may differ among species.

## Data accessibility

This article has no additional data.
